# Application to combine questioning, stimulus presentation and external measurements in a real-life music eye tracking experiment

**DOI:** 10.1016/j.mex.2024.102825

**Published:** 2024-06-25

**Authors:** Matthias Seitz, Miles Tallon, Karina Gotthardt, Katrin Rakoczy, Ulrich Frick

**Affiliations:** a Catholic University of Eichstätt-Ingolstadt, Ostenstraße 28a, D, Eichstätt 85072, Germany; b Research Centre, HSD University of Applied Sciences, Prüfeninger Str. 20, D, Regensburg 93051, Germany; c Research Centre, HSD University of Applied Sciences, Waidmarkt 3 und 9, D, Köln 50676, Germany; d Institute for Childhood and School Pedagogy, Justus-Liebig University Gießen, Karl-Glöckner-Straße 21B, D, Gießen 35394, Germany; e Institute for Clinical Epidemiology and Biometry, Julius Maximilian University, Josef-Schneider-Str. 2, D, Würzburg 97080, Germany

**Keywords:** Digital survey technique, Web app, Event driven immediate questioning, Digitalization, Questionnaire, Ambulatory assessment, Direct assessment, Integration of measurement tools, Eye-tracking, Synchronization, Remote control, Media control, Music psychology, Direct Assessment Survey (DAS)

## Abstract

Studying people in real-life situations, such as making music in a vocal group, requires flexible and integrative measurement technology. Therefore, a digital browser-based survey instrument was developed for this study. It was designed to seamlessly introduce questions on participants' mobile devices through external control immediately after relevant events, aiming to achieve high accuracy in self-administered situational questions. In addition, chronological synchronization with supplementary measurements (here eye-tracking and audio recording) was incorporated. Digital features of this web app offer convenient integration into everyday situations, synchronous interviewing of multiple people, and gathering time-based data. Due to the numerous possibilities of the browser-based development interface, various other application areas can be opened up. The contribution of this article is:

- App - explanation and offer for use
- Feasibility report on the implementation of the app in an eye-tracking study with vocal groups

## Graphical abstract

**Figure:**
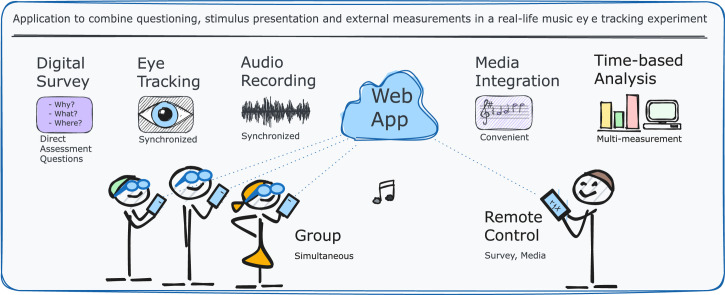

Specifications table

**Table:** 

Subject area:	Psychology
More specific subject area:	Measurement
Name of your method:	Direct Assessment Survey (DAS)
Name and reference of original method:	Ambulatory Assessment; Survey
Resource availability:	https://github.com/matthiasSeitz/directassessmentsurvey

**Method details**

## Introduction

The scientific investigation of creating music in a vocal group requires integrative and flexible measurement techniques to adapt to the dynamic process. Modern technologies nowadays can open up new and effective methods in musical research compared to earlier attempts. Therefore, in this pilot study, the survey technique was digitally implemented, inspired by contemporary principles such as ambulatory assessment [4] and other dynamic measurement applications [8]. The application was designed as a web app (demo version: [3]) for the purposes of allowing the experimenter to send questions or stimuli to all participants simultaneously at specific points in time, while allowing study participants to respond at their individual pace. The application was also designed for remote control of several groups of devices, such as the individual mobile devices of the participants and a common screen presenting visual stimuli for all participants. The digital survey application is enabled to record all data with timestamps, which allows synchronization with other measurement tools such as eye-tracking [6] or audio recording [7] and provides opportunities for time-based analyses methods such as failure time analysis (e.g., comparing response latency (cf. [9])). Presentation of stimuli or the timing of the questions were remotely controlled by the experimenter for simultaneity. The survey instrument is free of charge but requires mobile devices for participation and a computer or Web hosting that performs the server function.

This paper demonstrates the application and provides guidance to other researchers by describing all the steps necessary to use it themselves. We also describe the technical setup of the experiment, we explain the synchronization of the web app data with the eye-tracking and audio recordings, and we demonstrate a first step to validate the method.

## Description of the application

### Functionality and workflow during the experiment

The application was developed for an experiment in 2022, in which groups of three participants were examined as a singing group. Each participant was equipped with eye-tracking glasses, microphones and tablets running the application. The 30 min experiment contained different phases, namely playing instructional videos, singing together, evaluating direct assessment questions, completing questionnaires and eye-tracking validation. All of this was integrated with the application.

The application is browser-based and includes a specific function that enables the experimenter to display questions simultaneously on all participants' devices without the need for refreshing the browser window. This allows direct assessment questions (DAS-questions) to be asked immediately after fixed or even ad hoc defined steps of the workflow. This minimizes biasing memory effects and thus may result in a high reliability of the answers. For this purpose, the experimenter is provided with a control interface on a separate website. When the experimenter activates the respective step, the question is presented to all participants at the same time. As soon as a participant has completed his/her answers, he/she is forwarded to a waiting area. Once all participants are in the waiting area, the experimenter can forward them to the next phase of the experiment simultaneously. In addition to this function for DAS-questions, questionnaires can also be administered in a traditional manner, where participants can answer questions of multi-page questionnaires at their individual speed. The remote-control function of devices can be set up for several distinct device groups. For the study presented here, two device groups were set up for (1) mobile devices for each participant and (2) a screen visible for the whole experimental group. The mobile devices group was used to include the questionnaires, DAS-questions, musical notes, instructions and eye-tracking calibration markers on each participant's tablets. On the screen device group, all rehearsal videos for different songs, videos with standardized conducting for singers, playbacks, and eye-tracking calibration markers were included. All device groups were controlled via buttons on a separate website of the experimenter. Although the experimental set-up with eye-tracking of three persons, audio recording and interviews was very extensive, only one person had to be present to carry out the experiment due to the user-friendly operation of the digital application.

### Implementation

#### Description of operation

In this study, tablets, PCs, a TV screen, a Wi-Fi router, eye-tracking glasses, microphones, speakers and an audio interface were utilized. A LAMP stack [12] was set up on a PC to create a local network. This should achieve better performance than using a web space provider. The application was mainly written with PHP and JavaScript due to the simple development environment. A more detailed description than in the following section is available on Git Hub [11].

**Fig. 1:**
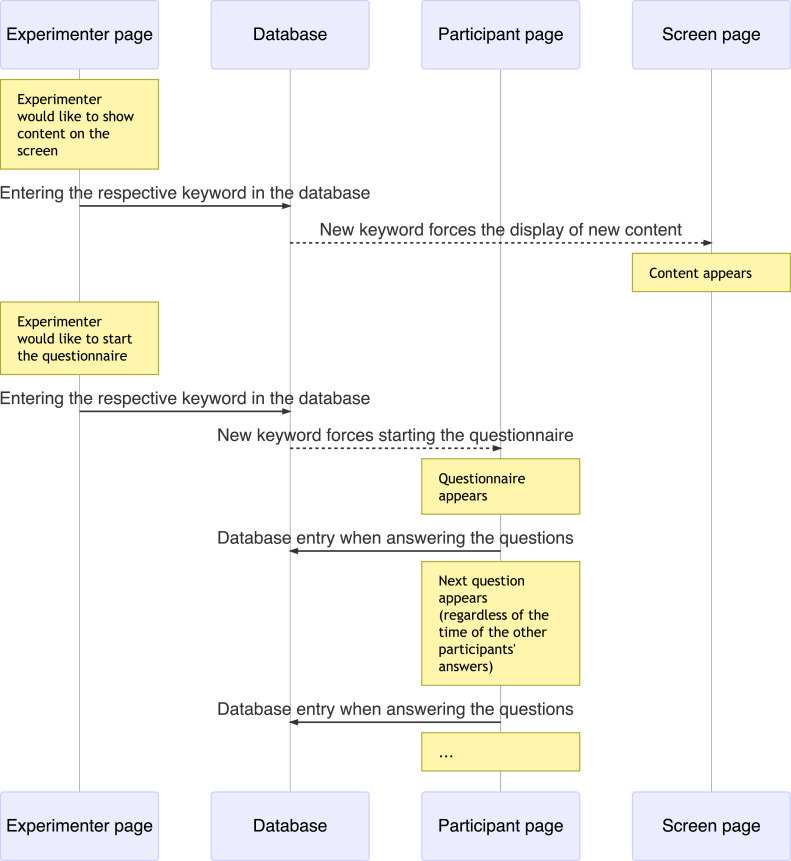
Example workflow of the application.

The application consists of separate websites that are assigned to the various requirements of different people or devices in the experiment. These are the participant page as an interface on mobile devices for each individual participant, the screen page for the large-scale display of content for the entire group, and the experimenter page for controlling the workflow of the study. The device pages (i.e. both the participant page and the screen page) can be controlled remotely by the experimenter via the experimenter page (see Fig. 1). The user interface of the experimenter page contains buttons for controlling the corresponding contents of the study on the device page, as well as further information such as a view of the other devices and information about the data already recorded. Each button creates a database entry using a keyword for the respective content. The device page checks for updates at a frequency of less than 1 Hz and compares the current keyword (stored in a session variable) with the latest keyword stored in the database. A new keyword found in the database is integrated into the device page and updates the display of new content. The keyword is the unique key for the content.

In addition to this remote control, the participants can also continue independently with questionnaires. In order to enable the questionnaires to be answered at the individual pace of the participants, each database entry (e.g. answer to a question) is assigned to the respective device that made it. By additionally filtering the database entries, it is possible to ensure both that all questionnaires can be completed at different pace and that the experimenter can remotely control the content presented.

#### Customization

A demo version [3] of the app is available on GitHub [11]. The device groups mobileDevices and bigScreen are already integrated in this demo version. The README.md file provides all the information you need to customize and use the demo version for researchers’ own projects.

The connection to your own database must be established, as well as the Internet address to your web space. Both will be included in the scripts where necessary. In the database a table must be created, which can be executed with the SQL command from the README file.

Start pages are linked to index.php, which provide form fields for participant usernames and session names. Username and session name are submitted to the mainPage (e.g. mobileDevices.php) and transferred to session variables for use during the session. Likewise, the logout.php file exists to automatically end the session with session_destroy after 120 s of inactivity. The automatic logout function is stored in the respective updatePages and can of course be adjusted or deleted.

All content (e.g., questions) to be integrated consists only of the code fragments of the content to be displayed instead of complete websites, as these are integrated into the code of the main pages via the PHP function include. These files are thus very simply structured and the creation of an individual survey can be easily implemented. All files of content to be included are stored in the parts folder.

To make a content (part) on the naviPage accessible as buttons, its file name (e.g. ``question_1.php'') must be included as naviKey via form for the database entry. To make this step as user-friendly as possible, the code elements that must be written before or after the respective naviKey have been saved as predefined variables at the beginning of the navigation page in the variables $form_pre and $form_post. Therefore, for each part to be included, only echo $form_pre . ``question_1.php'' . $form_post; needs to be written (here for question_1.php).

The appearance can, of course, be customized according to individual needs. In the file main.css the layout of the application can be defined.

### Feasibility report on the implementation of the app in an eye-tracking study with vocal groups

#### Eye-tracking settings

**Fig. 2:**
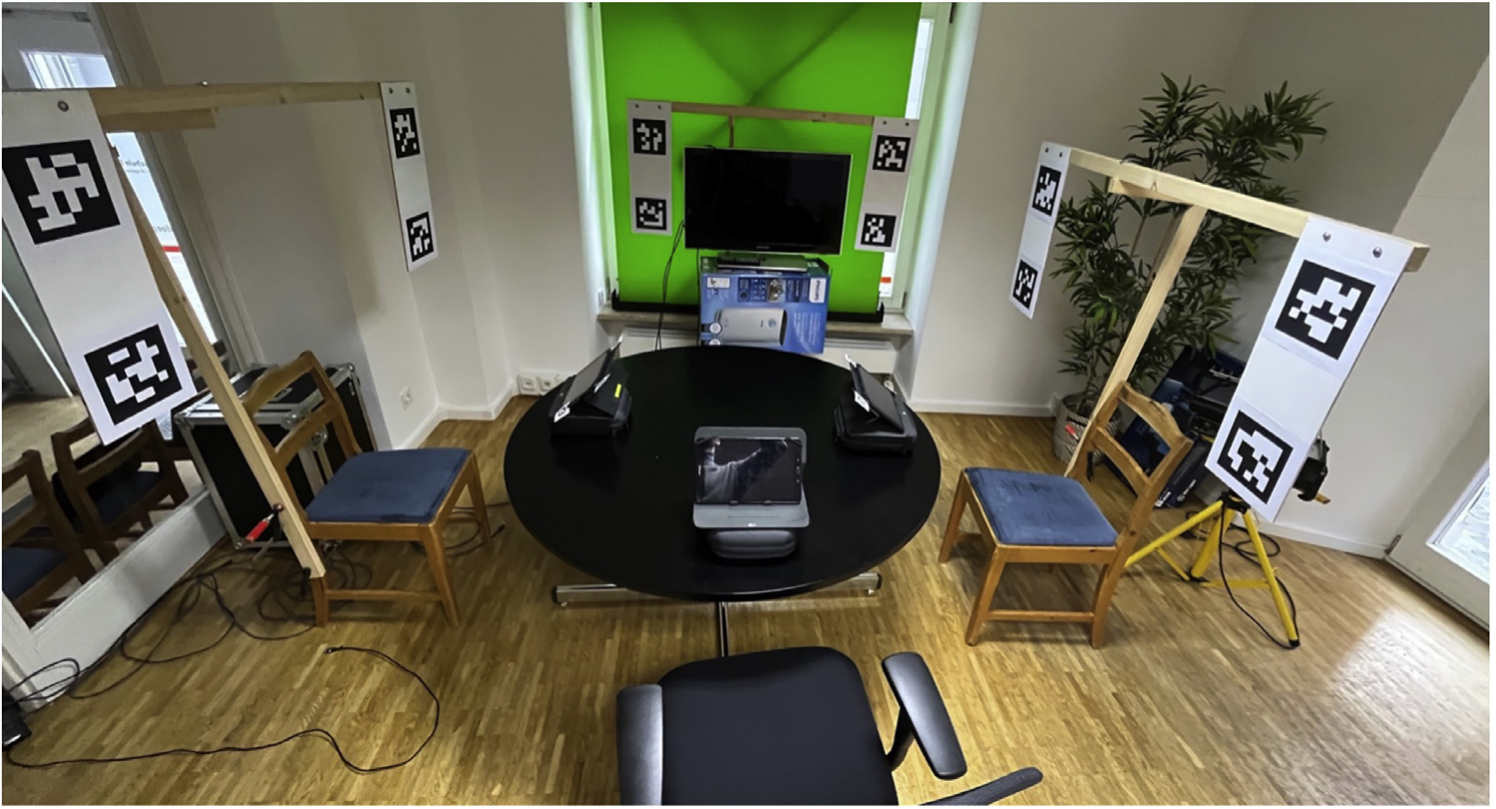
Setup of the experiment.

In this study, the participants' gaze behavior was recorded with eye-tracking glasses of the Pupil Labs Core brand [10]. In addition to conventional analyses of the eye-tracking method [5], the gaze behavior of the participants in three-dimensional space, in particular the eye contacts between all participants, was to be recorded. For this purpose, all relevant subjects and objects were each positioned within rectangular areas (surfaces) defined by four individually identifiable apriltags (similar to QR codes) of the 36 h11 series [1] attached to the outside corners of the rectangles (Fig. 2). A digital implementation of the apriltags on the screens mobileDevices and bigScreen would have been very elegant, but the recognition of the digital aptiltags by the glasses was significantly worse than in printed form. Wooden brackets were attached to the participants' chairs, framing an area around the participants' head and upper body, to provide an attachment of the apriltags for the area to be defined. The participants were seated at a circular table (diameter approximately 1.3 m) where the bigScreen was positioned at 12 o'clock and the participant at 3 o'clock (VP_right), 6 o'clock (VP_middle), and 9p'clock (VP_left). The maximum distance between eye-tracking glasses and apriltags in the experimental setup was therefore less than 2.5 m. The sizes of the apriltags were set to the values shown in Table 1 after previous testing based on the distances, angles, and lighting conditions of the setting.

**Table 1:** Hardware dimensions.

Surfaces	Surface height (cm)	Surface width (cm)	Apriltag side length (cm)
Participants (frame around head and shoulders)	49,5	70,0	15,0
Screen (flatscreen TV)	49,0	95,0	15,0
Tablets	15,0	26,0	3,0

The Pupil Labs Core eye-tracking glasses are equipped with two cameras that film the eyes of the participants (eyeCams) and with another camera that films the environment in the direction of the participant's gaze (worldCam). Data from the worldCam were recorded at maximum resolution (Pupil Cam1 ID2; 1920×1080px; fisheye; 155° horizontal; 85° vertical; 30 Hz) to take advantage of the full angle of the optical lens. This was important to ensure that all areas of interest of the experiment remained visible in the camera image at all times, despite lateral head movements of the participants. This is a prerequisite for the successful analysis of visual contacts between singers. The eyeCams were also recorded with the highest resolution (Pupil Cam2 ID0; 400×400px; radial; 39° horizontal; 39° vertical; 120 Hz). The glasses were calibrated using Marker v0.4_marker (Pupil Labs), first on the participants' tablets and then on the bigScreen visible to all. For calibration, the participants performed circular head movements while their eyes always fixed on the marker. The video data of the glasses were recorded on separate computers with the software Pupil capture (Pupil Labs capture version 3.5.1; compression: bigger files, less CPU).

### Audio/media settings

The audio recording of the experiment was done via individual microphoning of the two singing participants in order to obtain separate audio tracks for subsequent external analyses. For this purpose, Neumann KM184 microphones were pointed directly at the participants at a distance of about 50 cm and recorded uncompressed via an audio interface (Motu Ultralight Pro) using the software Reaper.fm. The audio recording was controlled directly and not via the application of the experiment. No other control was required because the audio recording could be started before the experiment began and ran continuously throughout the experiment.

All media content of the experiment was controlled by the developed application. Images and videos were displayed on the bigScreen and on the mobileDevices, and the sound of the acoustic stimuli was provided by two active Adam A5 speakers.

### Synchronization of survey data, eye-tracking videos and audio recordings

The synchronization of all data (eye-tracking videos, sound recordings, DAS-recordings, survey data) was performed subsequently using the timestamps of all recordings. The first video of the experiment was used as temporal reference for the synchronization. This video contained both a clearly defined acoustic onset and a simultaneous abrupt change of the image from dark to light. Using the visual reference, the temporal offset of the video recordings could be determined. All audio recordings could be synchronized based on the acoustic marker in the reference video. The temporal offset could be determined for all data and then used for the analysis of the data.

The accuracy of the synchronization resulted from the sample rate of the individual files. The frame rate of the reference video was 30fps, which corresponds to a frame interval of 33.3 ms. The synchronization of the video files of the eye-tracking glasses was based on this grid. The resolution of the audio tracks, on the other hand, is much finer. The sample rate of the audio material was 44.1 kHz, which corresponds to a sample interval of 0.023 ms. The synchronization of the audio tracks was based on this grid. The survey data could also be synchronized, since the playback of all videos (including the reference video) was done using the naviKey database entries of the experiment. Each database entry was stored with a stamp of the current time in milliseconds, which provides all the necessary values for synchronizing the data.

The method used is based on certain technologies that add different time delays (e.g. wireless LAN). However, the seamless integration of the applied technologies was essential in this real-life experiment and therefore such technical inaccuracies were accepted. With respect to the grid used (frame rate of the reference video: 33.3 ms) and the fixations of human gaze in the range of about 300 ms considered for the analysis, network-dependent delays within the locally used LAMP stack, as well as acoustic time delays were not considered relevant.

Checking the synchronization stability during the approximately 30 min experiment revealed constant synchronicity over the entire period of recordings.

The digital survey technology used in this study enables analyses on the basis of event times. This opens up further research possibilities compared to analog approaches. However, when analyzing collected data, synchronization must be considered in relation to the measurement accuracy of the technology used.

## Method validation

**Fig. 3:**
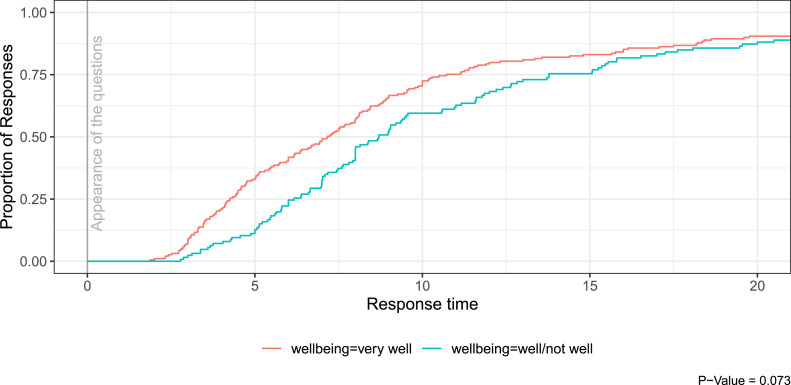
Response latency to direct assessment questions as a function of well-being.

The digitalization survey technique has the advantage that all answers can be analyzed according to the time of the entries by saving each measurement point. The following example from the experiment shows the answers of the participants to all DAS-questions in dependence of the self-reported well-being within the respective experimental group. The diagram shows that the questions were answered faster when the participants felt very well in the group. Well-being with the more reluctant classifications well/not well resulted in higher response delays. This can be regarded as a hint on the validity of the DAS-answers, as temporary self-evaluation is known to be connected to answering time [2] Fig. 3.

The use of the web App as a survey instrument and as a tool for displaying all media on the screens of the study was very helpful for feasibility. The intuitive controls with buttons on the navigation page of the web app allowed the experiment to be continued without delay. The possibility to fade in DAS-questions immediately after pre-specified events was essential for the study, because due to temporally varying phases of the experiment (a cappella singing, agreements, “real-life situation”) it was necessary to adapt the experiment's progression to differing lengths of working periods between and within experimental groups.

The questioning on mobile devices may be regarded as improving the accuracy of the measurement. This is plausible because the appearance of the questions with simultaneous fading out of the musical notes on the mobile devices intuitively prompted the participants to answer, for which verbal instructions would have been necessary with analog implementation. This immediacy of the survey conducted digitally also has the advantage of minimizing memory effects as well as eliminating most distractions that might otherwise occur after the event being evaluated.

Eye-tracking of the participants required careful administration in some cases. The mandatory and delicate adjustment of the cameras to the face of each participant, the preventing the glasses from slipping during the experiment, the wired fixation and last but not least the somewhat strange appearance with eye-tracking glasses may possibly influence the perception of the experimental setting. Nevertheless, the experiment could be carried out without any incidents and all data could be recorded.

The experiment in this study was conducted in a prepared room with a local network, LAMP stack and permanently installed devices. However, the flexibility of the developed application also allows applications outside the studio environment. In combination with domain and database of a web space provider, the application can be used in various social science studies in real-life surveys. The comfortable participation in surveys is made possible by the browser-based integration. Access to the survey is also facilitated by providing a QR code for direct linking to the survey page using the camera of the mobile devices. This function is already integrated in the demo version of the web app.

## Ethics statements

All participants gave informed consent to the experimental procedure and anonymized data storage. Ethical approval of this study was obtained from the HSD ethics committee (decision of April 27, 2022).

## CRediT authorship contribution statement

**Matthias Seitz:** Conceptualization, Methodology, Software, Formal analysis, Investigation, Resources, Data curation, Writing – original draft, Visualization. **Miles Tallon:** Conceptualization, Resources, Writing – review & editing, Visualization. **Karina Gotthardt:** Writing – review & editing. **Katrin Rakoczy:** Writing – review & editing. **Ulrich Frick:** Conceptualization, Methodology, Validation, Writing – review & editing, Supervision, Project administration, Funding acquisition.

## Declaration of competing interest

The authors declare that they have no known competing financial interests or personal relationships that could have appeared to influence the work reported in this paper.

## Data Availability

The data is already available on Git Hub.
